# Transcriptomic Analysis of Human Fragile X Syndrome Neurons Reveals Neurite Outgrowth Modulation by the TGFβ/BMP Pathway

**DOI:** 10.3390/ijms23169278

**Published:** 2022-08-17

**Authors:** Liron Kuznitsov-Yanovsky, Guy Shapira, Lital Gildin, Noam Shomron, Dalit Ben-Yosef

**Affiliations:** 1 Wolfe PGD Stem Cell Lab, Racine IVF Unit, Lis Maternity Hospital Tel-Aviv Sourasky Medical Center, Tel Aviv 64239, Israel; 2 Department of Cell and Developmental Biology, Sackler Faculty of Medicine, Tel-Aviv University, Tel Aviv 69978, Israel; 3 Sagol School of Neuroscience, Tel-Aviv University, Tel Aviv 69978, Israel

**Keywords:** Fragile X syndrome, human embryonic stem cells, neural differentiation, RNA sequencing, neurite outgrowth, TGFβ/BMP pathway

## Abstract

Fragile X Syndrome (FXS) is the main genetic reason for intellectual disability and is caused by the silencing of fragile X mental retardation protein (FMRP), an RNA-binding protein regulating the translation of many neuronal mRNAs. Neural differentiation of FX human embryonic stem cells (hESC) mimics the neurodevelopment of FXS fetuses and thus serves as a good model to explore the mechanisms underlining the development of FXS. Isogenic hESC clones with and without the FX mutation that share the same genetic background were in vitro differentiated into neurons, and their transcriptome was analyzed by RNA sequencing. FX neurons inactivating *FMR1* expression presented delayed neuronal development and maturation, concomitant with dysregulation of the TGFβ/BMP signaling pathway, and genes related to the extracellular matrix. Migration assay showed decreased neurite outgrowth in FX neurons that was rescued by inhibition of the TGFβ/BMP signaling pathway. Our results provide new insights into the molecular pathway by which loss of FMRP affects neuronal network development. In FX neurons, the lack of FMRP dysregulates members of the BMP signaling pathway associated with ECM organization which, in a yet unknown mechanism, reduces the guidance of axonal growth cones, probably leading to the aberrant neuronal network function seen in FXS.

## 1. Introduction

Fragile X Syndrome (FXS) is the most common form of inherited intellectual disability. It is caused by inactivation of the *FMR1* gene and its encoded protein FMRP due to a CGG-repeat expansion in the 5′-untranslated region of the gene (when over 200 repeats), leading to DNA hypermethylation-mediated *FMR1* silencing [1]. FMRP is an RNA-binding protein that regulates mRNA transport, stability, and translation [2]. In accordance, the absence of FMRP in FX results in altered patterns of protein synthesis [3,4,5], which leads to impaired signaling in a number of intracellular pathways involved in neural differentiation and maturation [6,7]. This altered protein translation eventually leads to damaging neuronal network activity throughout the brain, which is critical for learning and memory [8].

Several in vivo and in vitro models are being used to investigate FX pathologies. *FMR1* knockout (KO) models were previously generated, mainly in mice [9,10] but also in zebrafish and drosophila [11,12]. However, there are many substantial differences between the human brain and that of animal models. The human in vitro models available are post-mortem adult neurons or neural precursor cells (NPCs) extracted from aborted fetuses [7,13,14,15] and induced pluripotent stem cells (iPSCs) generated from FXS individuals [16,17], but none of these human in vitro models express *FMR1*. In vitro neural differentiation of human embryonic stem cells (hESCs) can generate NPCs and cortical neurons that resemble fetal neurons [18,19], imitate human development, and thus serve as a powerful model to investigate the initial stages of neurodevelopment in FXS. Thus far, we derived several male FX-hESCs lines from FX-affected blastocysts, carrying the full natural mutation of the *FMR1* gene [20,21]. Using this paradigm to study FX, we now know that *FMR1* engages in neural progenitor differentiation, neuronal maturation, and synaptic network function [22,23,24,25,26,27]. However, it is not yet fully understood which molecular pathways are expressed at the initial stages of neurogenesis and are later translated to the impaired FX neuronal phenotype.

Although most research into the neurologic role of FMRP is so far directed at mature neurons, there is a growing body of evidence to suggest that at least some of the deficiencies characterizing FXS are due to aberrant development that accompanies the gradual inactivation of FMRP during embryonic development. Neural differentiation protocols give rise to highly heterogeneous cellular populations with distinct cell-fates, including NPCs, neurons, and glia. A number of studies, including ours, revealed a significant reduction in *FMR1* expression already in hESC-derived NPCs, leading to disparate expression of key neurodevelopmental genes that are shown to regulate proliferation, survival, and differentiation, highlighting the role of FMRP during neurogenesis [27,28,29].

We used isogenic sub-clones from a parental hESC line carrying the full *FMR1* mutation (>200 CGG repeats) and an isogenic control clone free of the *FMR1* mutation that share the same genetic background, together with an accelerated gradual neural differentiation protocol that mimics the natural process of the disease in vitro. This multi-tiered approach was used together with RNA sequencing and migration bioassay to further decipher the molecular and cellular basis underlying the aberrant function of FX neurons.

## 2. Results

### 2.1. Delayed Neurodevelopment of FX-hESCs

**Figure 1:**
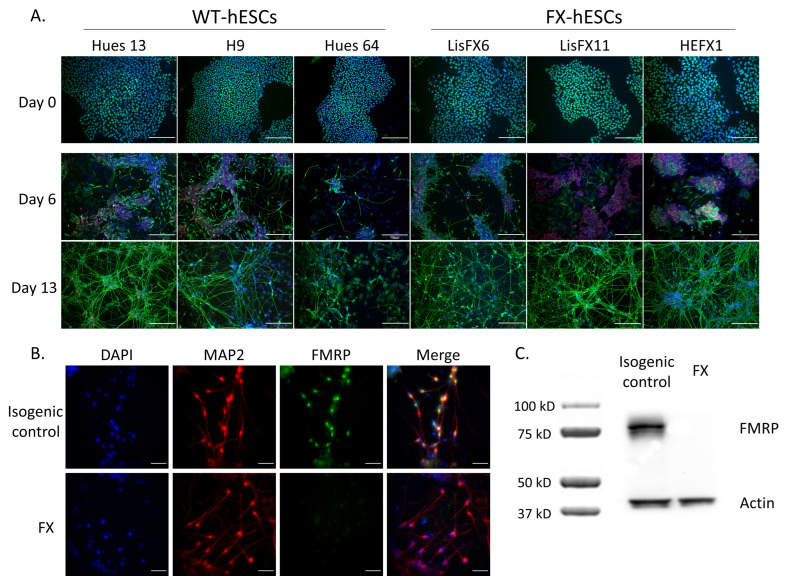
Delayed early neurodevelopment is concomitant with *FMR1* silencing. (**A**) Immunofluorescence staining of three FX- and three WT-hESC lines at 0, 6, and 13 DIV of accelerated neural differentiation, with the pluripotent marker POU5F1 (OCT4, green) and the neural genes β-III tubulin (Tuj1) (green) and the neural precursor marker PAX6 (red). Cell nuclei were stained blue by DAPI. Scale bar: 200 µm. (**B**) Immunofluorescence staining of a full-mutation sub-clone and its isogenic control for microtubule-associated protein 2 (MAP2) (red) and for FMRP (green), at 13 DIV. Cell nuclei were stained blue by DAPI. Scale bar: 50 µm. (**C**) Western blot analysis of FMRP expression in 23 DIV neurons following magnetic sorting (MACS) with CD184 antibody (negative fraction). Actin served as a positive control.

Using an improved and accelerated in vitro neural differentiation protocol, including a combinatorial application of six signaling pathway inhibitors, to robustly convert human pluripotent stem cells into a population of post-mitotic cortical neurons, a large number of neural cells expressing different neuronal genes were successfully generated from all three control hESC lines (Hues 13, H9 and Hues 64), as well as from all three FX-hESC lines (LisFX6, LisFX11 and HEFX1). A dense population of PAX6 and CNS neural precursors was generated already within 6 days in vitro (DIV); and within 13 DIV, approximately 50% of the cells expressed the neuronal gene Tuj1^+^ (Figure 1A). The FX-hESCs lines demonstrated delayed differentiation with more PAX6^+^-NPCs after six DIV as compared to the control, and less early born neurons (Tuj1^+^) were generated. However, since the use of isogenic hESC lines is important when focusing on the effect of *FMR1* inactivation solely while reducing the effects of other genes that can emerge when cells with different genetic backgrounds are compared, in all further experiments, isogenic sub-clones that were recently isolated in our lab were used: one in the full-mutation range (LisFX6 FX clone), and an isogenic control that is free of the mutation (<50 CGG repeats) [24]. Both the FX clone and its isogenic control stained positive for the pluripotent markers OCT4, SSEA4, and TRA-1-60, but only the FX clone inactivated *FMR1* expression following neuronal differentiation (Figure 1B,C), mimicking the natural *FMR1* silencing characterizing FXS fetuses at early development.

**Figure 2:**
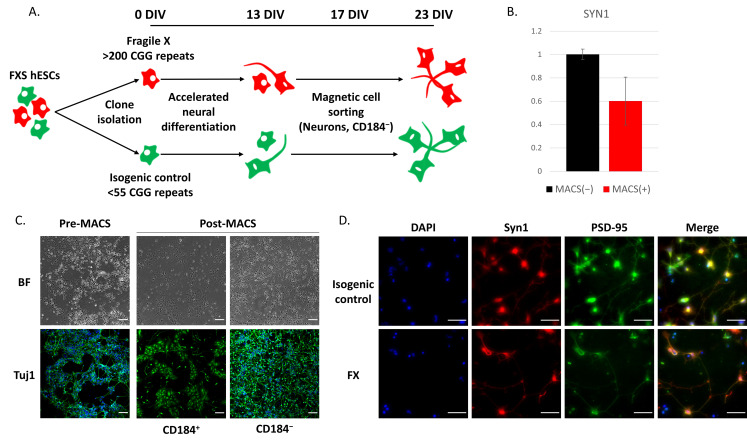
Enrichment of neuronal population following neural differentiation of hESCs. (**A**) Experimental scheme of generating FX and isogenic control neurons, differentiation, and magnetic sorting for neuronal enrichment with the cell surface marker CD184. (**B**) RNA expression of synapsin-1 (SYN1) by qRT-PCR at 23 DIV following MACS of isogenic control cells. The positive fraction served as control to normalize the values obtained. The housekeeping gene GAPDH served as an internal control. Three independent biological experiments were performed, and values are presented as mean± Standard error. (**C**) Neural cells at 23 DIV before and after MACS with CD184. Bright field (BF) and immunofluorescence staining for the neuronal gene Tuj1 (green). Cell nuclei were stained blue by DAPI. Scale bar: 100 µm. (**D**) Immunofluorescence staining of FX and its isogenic control cells following in vitro neural differentiation and neuronal enrichment, for SYN1 (red) and for the postsynaptic density protein 95 (PSD-95; green). Cell nuclei were stained blue by DAPI. Scale bar: 50 µm.

Following in vitro neural differentiation, various cell types are generated, including NPCs, neurons, and glia. In order to examine gene expression effects of FX specifically in neurons, we enriched their population in the culture by deselecting glia and NPCs expressing the cell surface marker CD184 using magnetic cell sorting (MACS; Figure 2A). Plating the cells following their sorting resulted in an enrichment of mature neurons expressing synapsin-1 (SYN1) within the CD184 negative fraction (Figure 2B). These neurons stained positive for the neuronal marker Tuj1 (Figure 2C) and the synaptic genes SYN1 and PSD-95 (Figure 2D), demonstrating their synaptic maturation. This enriched neuronal population will enable further examination of how a lack of FMRP affects gene expression in neurons, without confounding effects resulting from other neural cells comprising the cell culture.

### 2.2. Loss of FMRP Leads to Altered Gene Expression throughout Neuronal Differentiation

**Figure 3:**
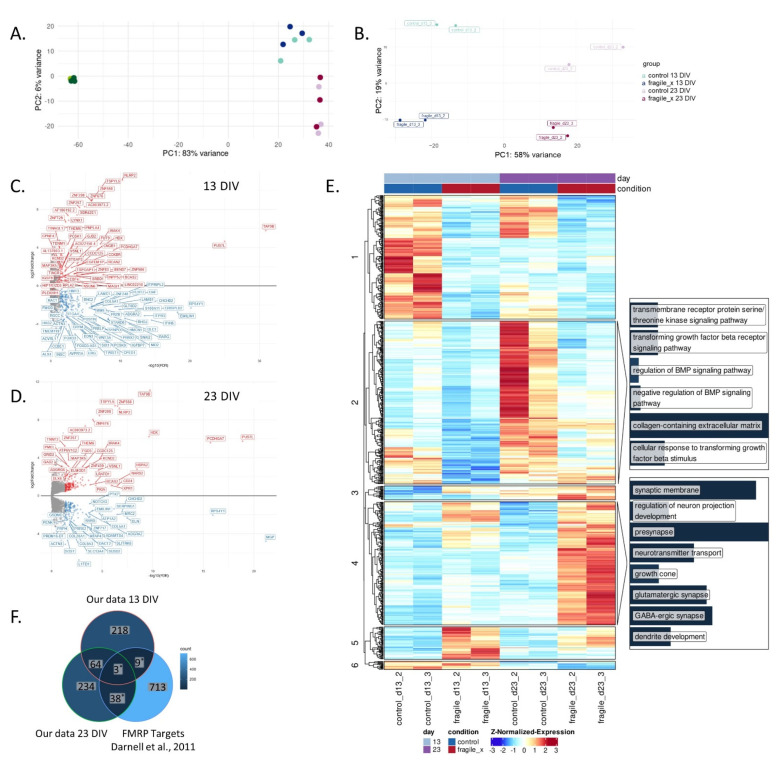
Transcriptomic analysis of neurons derived from FX and isogenic control hESCs. (**A**,**B**) Principal component analysis (PCA) plot of RNA-seq data. Colors represent cell line and DIV: d0 (hESCs), d13 (NPC and early neurons) and d23 (enriched population of neurons following MACS with CD184; (**A**) PCA of 0, 13, and 23 DIV during neural differentiation of hESCs; (**B**) PCA of 13 and 23 DIV samples. (**C**,**D**) Volcano plots of DEGs in FX compared to isogenic control, on 13 (**C**) and 23 DIV (**D**) of neuronal differentiation. Significantly downregulated genes relative to control are shown in blue and upregulated genes are shown in red. FDR < 0.05. (**E**) Heatmap of differentially expressed genes (DEGs) at 13 and 23 DIV of FX- and isogenic control-hESCs. Columns represent samples and rows represent genes, clustered by expression patterns, with added enriched gene ontology (GO) terms of some clusters. The heatmap is colored according to normalized, Z-transformed gene expression values, ranging from lowest (blue) to highest (red). Gene enrichment bars contain only significantly over-represented terms within the respective gene cluster, with bar height representing -log10(qvalue) (adjusted significance of enrichment). (**F**) Venn diagram showing the overlap of DEGs identified in this study (13 and 23 DIV) and FMRP targets published in Darnell et al., 2011 [3] (* hypergeometric test; *p* < 8 × 10^−6^).

To further determine *FMR1*’s role in neural maturation, RNA sequencing was performed on samples extracted from different time points during neuronal differentiation of both FX and isogenic control cells. Bioinformatic analysis identified a distinct transcriptomic signature for each time point, most notably for the pluripotent hESCs (0 DIV) compared to that of neurons (13 and 23 DIV), in both FX and control cells, accounting for over 80% of the total variance per principal component analysis (PCA; Figure 3A). PCA analysis of RNA-seq data show that FX and isogenic control cells present some similar transcriptomic signatures at each differentiation stage, demonstrating that these are indeed isogenic lines originating from the same parental line, and thus share the same genetic background. The distinct gene expression patterns in the three time points in both FX and its isogenic control cells confirm successful neuronal differentiation, and they demonstrate that the absence of FMRP does not affect their pluripotency as well as their differentiation potential. It was interesting to realize that, although all cultures looked sterile through all stages of differentiation, even following a very thorough examination under the microscope, viral RNA was found in two of the samples (i.e., d13 control and fragile X; experiment #1; Figure S1A), coinciding with expression of immune-response genes and causing a skew in gene expression that persisted to 23 DIV. Samples affected by the contamination were therefore excluded from further analysis (Figure S1B,C).

The PCA results show that, following 23 DIV, the FX cells clustered closer to their 13 DIV control counterparts (Figure 3B), indicating a delayed differentiation of the FX cells, as also shown by immunostaining (Figure 1A). A total of 294 significant differentially expressed genes (DEGs) were identified in FX d13 compared to its isogenic control (FDR < 0.05; Figure 3C), and 339 DEGs in d23 (FDR < 0.05; Figure 3D). Gene-Ontology (GO) enrichment analysis of these DEGs showed enrichment in pathways related to the regulation of the bone morphogenetic protein (BMP) signaling pathway, cellular response to transforming growth factor beta (TGFβ) stimulus, and extracellular matrix (ECM) organization (Figure 3E). Other enriched pathways were related to dysregulation of neurogenesis and include neuron migration, axonogenesis, axon guidance, glutamatergic synapse, and GABA-ergic synapse, (Figure 3E, cluster 4). More importantly, a significant number of the DEGs identified in this study were previously identified as mRNA targets of FMRP in the mouse brain [3] (Hypergeometric test *p* < 8 × 10^−6^; Figure 3F). These results suggest that the TGFβ/BMP pathway is probably regulated by FMRP to govern the organization and development of neural cells. Consequently, FMRP inactivation in FXS and autism spectrum disorders is correlated with aberrant development of the central nervous system.

### 2.3. Fragile X-Derived Neurons Display a Neurite Outgrowth Defect Manifested by the BMP Pathway

**Figure 4:**
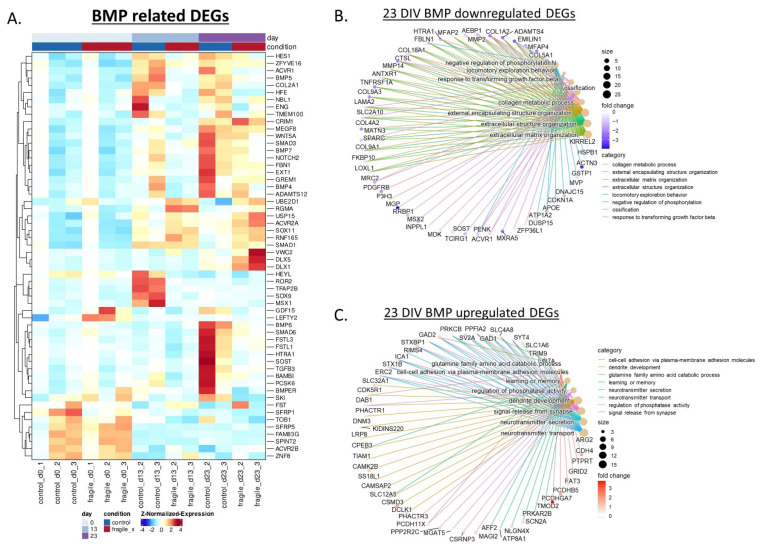
BMP related genes are involved in the altered differentiation of FX neurons. (**A**) Heatmap of DEGs related to the BMP pathway during neuronal differentiation of FX- and isogenic control-hESCs (13 and 23 DIV). Columns represent samples and rows represent genes, categorized into clusters of common expression patterns. The heatmap illustrates lower (blue) to higher (red) gene expression levels. (**B**) A GO enrichment plot of 23 DIV downregulated DEGs, with a focus on BMP. (**C**) A GO enrichment plot of 23 DIV upregulated DEGs, with a focus on BMP.

We previously showed that FX NPCs demonstrate abnormal neurite outgrowth [18], but it is not yet clear which molecular pathways are involved and how neurons are affected. The GO enrichment analysis of our DEGs by clusters of common expression patterns (co-expression), highlights cluster 2, in which there is an enrichment for genes involved in the BMP signaling pathway (cluster 2 in Figure 3E; Figure 4A). In our dataset, altered expression of BMP signal transduction genes is evidenced by 13 DIV of neural differentiation with mainly inhibitors of the BMP pathway. At 23 DIV, the downregulated BMP genes were also largely associated with ECM organization, as seen by the GO enrichment analysis where many genes are commonly connected to the BMP pathway and the ECM (Figure 4B). On the other hand, the upregulated BMP genes were also associated with regulation of neuron projection development, dendrite development, neurotransmitters secretion, and signal release from synapse (Figure 4C).

**Figure 5:**
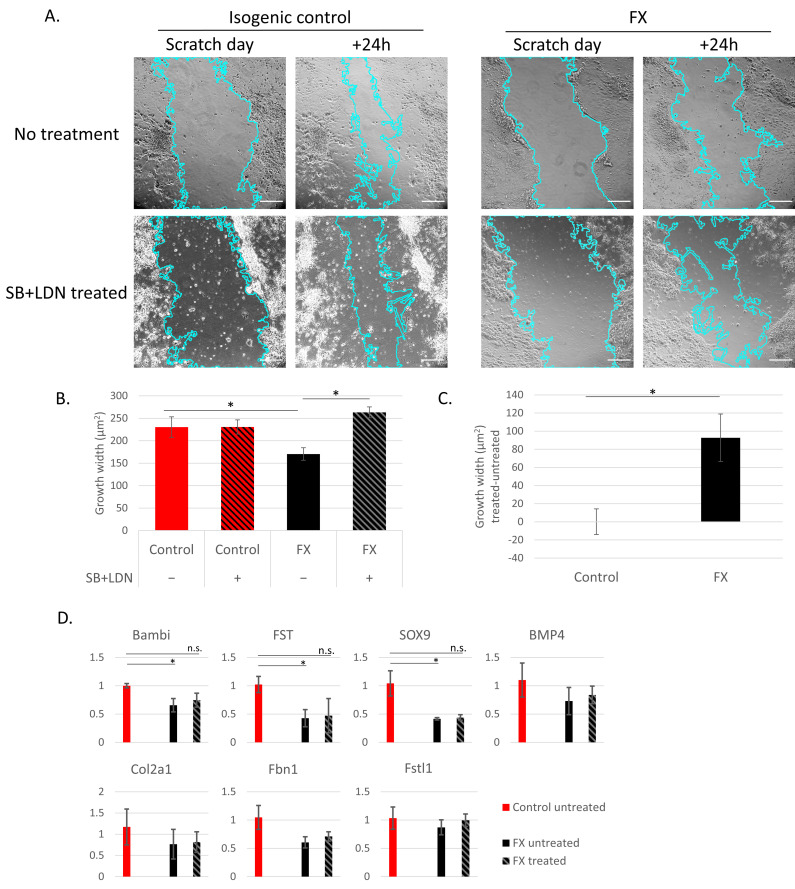
Fragile X-derived neurons display a neurite outgrowth defect. (**A**) Representative images of isogenic control (left) and FX (right) clones, after the scratch and 24 h following it, with (upper) or without (lower) SB431542 and LDN-193189 (10 µM and 250 nM, respectively). Surface area analyses was measured using ImageJ software 1.53f51 (NIH, http://rsbweb.nih.gov/ij/, USA). Scale bar: 200 µm. (**B**) Quantified values are growth width average calculated as the difference between the averaged cut width at the cutting time and the width left after 24 h. All values are mean ± SEM (* *p* < 0.0001; One-way ANOVA). Data obtained of each clone from three independent experiment and 30 fields, or more, were analyzed in each group. (**C**) Quantification of the difference in growth width between the treated and untreated cultures (* *p* < 0.05; Paired Student’s *t*-test). (**D**) RNA expression of DEGs from the BMP pathway analyzed by qRT-PCR at 14 DIV, in FX and control cells after a day with or without treatment of the TGFβ/BMP/SMAD inhibitors, SB431542 and LDN-193189. The untreated isogenic control served as a negative control to normalize the values obtained. The housekeeping gene, GAPDH, served as an internal control. Three independent biological experiments were performed, and values are presented as mean± Standard error. * *p* < 0.05, *t*-test.

Neurite outgrowth is an important process during normal early neurodevelopmental which regulates the proper axons and dendrites formation and eventually leads to the development of synaptic connections. In order to explore neuronal migration in a functional in vitro bioassay a scratch assay was conducted at 13 DIV, and the neuronal extensions were measured and quantified. The results show decreased neurite outgrowth in FX neurons compared to their isogenic controls (Figure 5A; no treatment). The addition of inhibitors of the TGFβ/BMP/SMAD signaling pathway, SB431542 and LDN-193189, rescued FX cells by significantly increasing their neurite outgrowth to a level similar to the control (Figure 5B), while the same treatment had no effect on outgrowth of control neurons (Figure 5C). qRT-PCR demonstrated that this rescue in neuronal function upon treatment with BMP inhibitors is correlated to significant correction in the expression of several DEGs related to the BMP pathway (Figure 5D).

**Figure 6:**
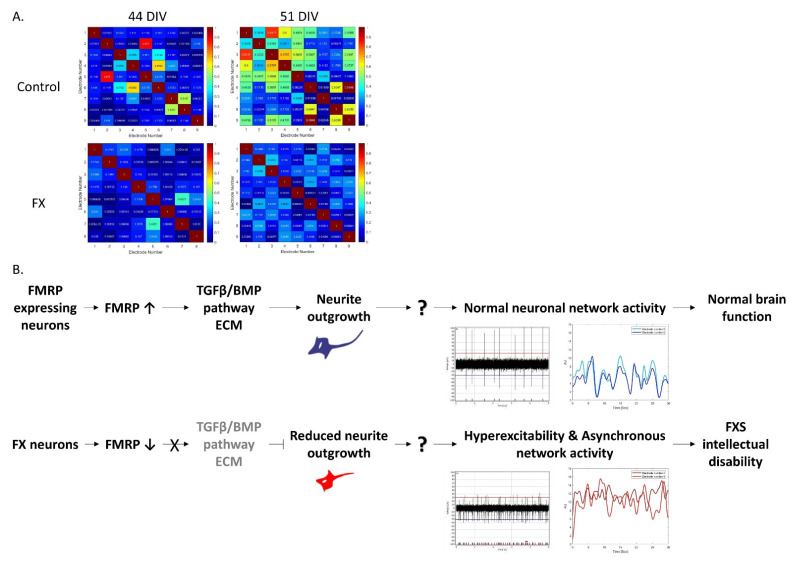
Suggested mechanism by which *FMR1* inactivation regulates the impaired functional connectivity underlying Fragile X syndrome. (**A**) Neurons were plated, measured, and analyzed by multielectrode array recordings and data analyses were performed as previously described in Gildin et al., 2022 [24]. These heatmaps summarize cross-correlation among all multielectrode array active electrodes (≥15 spikes/min) of WT (top) and FX (bottom) derived neurons at 44 DIV (left) and 51 DIV (right). The panel ranges from high synchrony/high cross-correlation among neurons (red), to low synchrony/low cross-correlation (blue). Diagonal red patterns stand for autocorrelation within the recorded neurons. (**B**) Schematic presentation of the suggested molecular pathway regulating aberrant function in FXS. In control neurons, FMRP is important for the regulation of BMP signaling pathways and ECM molecules expression: these in turn control proper neurite outgrowth. Together with the formation of normal neuronal network activity, learning and memory abilities are generated. In contrast, in FX-derived neurons, FMRP is not expressed, leading to altered expression of BMP and ECM signaling pathways and resulting in reduced neurite outgrowth. Eventually, with the hyperexcitable and less synchronous neuronal networks, intellectual disability is formed.

We recently demonstrated that impaired functional connectivity underlies Fragile X syndrome, in which FX neuronal networks are more hyperexcitable and less synchronous than that of the control [24]. By differentiating FX-hESCs even longer, through 51 DIV, we showed that this feature of asynchronous network activity in FX neurons is even more pronounced (Figure 6A). Together with the data presented in this study, we suggest the following molecular mechanism to regulate the aberrant function of FX neuronal networks (Figure 6B). In FMRP-expressing neurons, the regulation of the TGFβ/BMP pathway allows for normal neurite outgrowth and axonogenesis, resulting in good neuronal network activity and leading to the development of normal learning and memory abilities. In contrast, in FX neurons, the lack of FMRP changes the local expression of different members of the BMP signaling pathway that reduces the guidance of axonal growth cones. Together with the hyperexcitability and asynchronous neuronal network activity, this mechanism of action can explain the origin for development of intellectual dysfunction associated with FXS.

## 3. Discussion

In this study, we used a set of isogenic hESC subclones of full-mutation FX and control lines that share the same genetic background, together with an accelerated in vitro neuronal differentiation protocol, followed by neuronal enrichment, in order to investigate the molecular alterations controlled by *FMR1* inactivation. RNA sequencing analysis demonstrated that, already at the early stages of neurodevelopment, the FX neural cells present altered and delayed maturation, which may affect the electrophysiological function of the mature neurons within the brain. We identified changes in the TGFβ/BMP signaling pathway: these DEGs were also associated with ECM organization and neuronal migration. We found that the neurite outgrowth of FX neurons is decreased, which was corrected by inhibiting the TGFβ/BMP/SMAD signaling pathway, showing its impact on the abnormal neurodevelopment induced upon FMRP loss.

FMRP is highly expressed in healthy individuals during early embryogenesis and is required for proper neuronal differentiation [7,30]. It is an mRNA-binding protein which regulates the translation of hundreds of proteins [3]. In order to mimic the effect of FMRP silencing on the embryo’s neurodevelopment, the accelerated neural differentiation protocol was applied in this study, which generated a heterogenous neural cell population, including astrocytes that autonomously support even very long neuronal cultures. Since the focus of this study was the neurons, and in order to reduce variations among experiments, the neuronal population was enriched using MACS to gently exclude NPCs and glial cells from the analyzed cultures. A similar gene expression profile was observed in both FX and their isogenic control hESCs indicating that, although the FMRP mutation does not harm the differentiation potential of the cells, it does disrupt neuronal related molecular pathways. The association between FMRP silencing and the aberrant gene expression profile is reflected in the DEGs wefound, which were enriched in FMRP targets also previously found in FX-mouse brains [3]. The RNA sequencing analysis also demonstrated alterations in pathways related to the ECM, neurite outgrowth, synaptogenesis, and synaptic function and maturation, that collectively affect learning and memory capabilities associated with FXS [31,32]. Altogether, these results suggest that the absence of FMRP leads to dysregulation of critical processes in the developing brain that consequently probably contribute to the cognitive deficiencies underlying FXS.

The TGFβ/BMP signaling pathway is tightly engaged in early neurodevelopment when BMP ligands and receptors are expressed in a complex manner in all regions of the central and peripheral nervous system [33,34,35]. The strict regulation of this pathway controls cell fate specification and maturation. Among its roles in neurogenesis is the regulation of dendritic development, neurite outgrowth, axon growth, and synapse formation, plasticity, and transmission [33,36]. In the present study, we show the involvement of TGFβ/BMP signaling in the disrupted development of FX neurons, as well as the dysregulation of neuronal maturation and synaptic genes. It was previously shown in neurons derived from *FMR1*-KO mice and FXS patients that FMRP binds the BMP type II receptor (BMPR2) and regulates its downstream targets, leading to abnormal synaptogenesis [37]. These results are in accordance with those found in the *Drosophila* FX-KO model in which its locomotion abnormality was reversed by inhibiting the BMP downstream target, LIMK [38]. Aberrant expression of the BMP pathway was also found in other neurodevelopmental and neurodegenerative disorders, such as Angelman syndrome, Alzheimer’s disease, and Huntington’s disease [33]. All these results, together with those found here for human FX neurons, points out at the extensive involvement of the FMRP-regulated BMP pathway in neurogenesis.

We and others previously reported defective neurite outgrowth and downregulation of axon guidance genes in neurons derived from FX- human pluripotent stem cells [17,18,26]. Other studies also showed abnormalities in neural development, abnormal dendritic spine morphology, and deformed growth cone development that affect axon guidance, regulated at least in part by the BMP pathway [39,40,41,42]. Here, we show for the first time that the reduction in neurite outgrowth in the early development of FX-derived neurons can be corrected by the inhibition of the TGFβ/BMP signaling pathway. The dynamics and stabilization of actin and microtubules is a major factor in neurite outgrowth and is regulated by BMPs [43,44]. Our differential gene expression analysis identified downregulation of inhibitory genes of the TGFβ/BMP signaling pathway, such as *BAMBI*, *FST,* and *FSTL1*. Since it was shown that neurite outgrowth is altered in a concentration-dependent manner while high concentration impaired their growth and low concentrations promoted neurite outgrowth [45], we assume that the balance of activation–inhibition is disrupted. Upon FMRP absence, the inhibition of the TGFβ/BMP signaling pathway is dysregulated, leading to decreased neurite outgrowth. By inhibiting BMP signaling, we were able to correct the FMRP-related neuronal phenotype of neurite outgrowth. Together, these results demonstrate the dynamic role of BMP as a morphogen and underlines its importance in neurogenesis and in the pathogenesis of FXS neuro-phenotypes. Further studies are needed in order to explore the distinct components of the BMP pathway that are involved in regulating axonal guidance and proper neuronal network maturation and activity in FXS.

Our results show the delayed maturation of FX-derived neurons at the transcriptomic level and at the functional level by the retarded neuronal outgrowth and maturation, pointing at related impaired neurodevelopmental pathways: mainly the TGFβ/BMP signaling pathway. Furthermore, we propose that this impaired gene expression is probably one of the reasons for the less synchronous neuronal network activity we recently observed in FX neurons [24], eventually leading to intellectual disability. Processes impaired by FMRP downregulation already at early stages of development can explain the aberrant functional activity of their derived neurons arising later in development, such as hyperexcitability and asynchronous neuronal networks, promoting the importance of neural differentiation protocols that mimic the biological progress of the syndrome in the human fetus. These results highlight the value of studying isogenic hESC lines when investigating the downstream regulators of FMRP on differentiating neurons. Gaining better understanding of these regulating mechanisms will provide new therapeutic targets for FXS. Although animal models can also be used for discovering potential new drugs for treating FXS, it is well accepted that, before starting clinical trials with human patients, potential drugs should be tested on human pre-clinical experimental models. Some molecular pathways are also unique to human physiology. Therefore, these hESC-derived neurons can serve as a great platform for drugs screening and discovery for neurodevelopmental diseases in general and for FXS in particular.

## 4. Materials and Methods

### 4.1. Human Embryonic Stem Cell Culture

The use of spare in vitro fertilization (IVF)-derived embryos following preimplantation genetic diagnosis (PGD) for the generation of hESCs was approved by the Israeli National Ethics Committee (7/04-043), and in accordance with the guidelines released by the Bioethics Advisory Committee of the Israel Academy of Sciences and Humanities. All experiments were conducted using a pair of FX and its isogenic control sub-clones that were derived from the original Lis_FX6 hESC line of relatively early passage (*p* 40–55) that present CGG repeats from the normal to the full mutation range. Clone 8A has >200 CGG repeats, and thus served as the full mutation clone: clone 7B has <50 CGG repeats, and thus served as its isogenic control that shares the same genetic background. Full characterization of these isogenic clones is described in Gildin et al. [24], including CGG repeats number analysis by a specific designed PCR CGG repeat number assay and by the AmplideX PCR/CE *FMR1* Reagents (Asuragen, Austin, TX, USA), in which confirmation of their polymorphic markers by CA repeats analysis are known to identify their parental Lis_FX6 line, and the expression of pluripotent markers. In addition, three male FX-hESC lines derived in our lab were studied: LisFX6 [21,46], HEFX1 [20,47], and LisFX11 (see characterization in Figure S2). The following control hESC lines free of *FMR1* mutation were also used: HUES-13 and HUES-64 ([48,49]; kindly provided by Dr. Douglas Melton, Harvard University) and H9 ([50,51]; WiCell, University of Wisconsin). hESCs were treated as we previously described [24].

### 4.2. In Vitro Neural Differentiation

hESCs were differentiated into cortical neurons by an accelerated dual SMAD inhibition protocol as previously described [52,53]. Briefly, hESCs were plated on Geltrex with mTeSR1 to confluence. From day 0, cells were cultured in KSR media in the presence of small chemical inhibitors of the TGF, SMAD, and Wnt pathways until six DIV. From two DIV, cells were cultured also in the presence of MEK, FGF, and Notch signaling inhibitors to trigger cortical precursors for cortical neuron differentiation. N2/B27 medium was added in increasing 1/3 increments every other day from four DIV, until reaching 100% Neurobasal at eight DIV. The WNT agonist CHIR99021 was added at 8-15 DIV, as it exerted a strong pro-survival effect on cultures. After MACS, cortical neurons were cultured on Poly-L-ornithine hydrobromide/Laminin I/Fibronectin coated wells in NB/B27 supplemented with 1% Pen/Strep (03-033-1B, Biological Industries, Beit Haemek, Israel), BDNF, dbcAMP, and ascorbic acid.

Coating plates for replating with Poly-l-ornithine/Laminin I/Fibronectin: plates were coated with 15 μg/mL Poly-l-ornithine (P4957, Sigma-Aldrich, St. Louis, MI, USA) in PBS and incubated over night at 37 °C in 5% CO2. The next day, Poly-L-ornithine was removed, washed with 1X PBS, and plates were coated with 1 μg/mL Laminin I (L2020, Sigma-Aldrich) and 2 μg/mL Fibronectin (F1056, Sigma-Aldrich) in PBS, air dried for 45 min at room temperature, and kept at 4 °C.

### 4.3. Immunofluorescence

Cells were fixed with 4% paraformaldehyde (PFA; P6148, Sigma-Aldrich) for 20 min at room temperature (RT). Blocking was performed with blocking solution: 10% Fetal Bovine Serum (FBS) or 5% Goat serum (GS) with 0.2% Triton X 100 in PBS for permeabilization. Cells were then incubated with primary antibodies (anti-SSEA4, CST-4755, Cell Signaling Technology, Danvers, MA, USA; anti-TRA-1-60, ab16288, Abcam, Cambridge, UK; anti-OCT4, sc-5279, Santa-Cruz, Starr County, TX, USA; anti-FMRP, BLG-834601, Biolegend, San Diego, CA, USA; anti-Tuj1, BLG-801201, Biolegend; anti-PAX6, BLG-901301, Biolegend; anti-MAP2, sc-20172, Santa Cruz; anti-SYN1, AB1543, Merck, Darmstadt, Germany; anti-PSD-95, MAB1596, Merck) diluted in blocking solution for 1 h at RT, washed 3 times with PBS, and incubated with secondary antibodies (donkey anti-mouse Alexa Fluor 488, A21202, Thermo Fisher Scientific, Waltham, MA, USA; goat anti-rabbit Alexa Fluor 594, A11012, Thermo Fisher) for another 1 h at RT in the dark, and counterstained with DAPI for nucleus localization (D1306, Thermo Fisher Scientific). Cells were mounted with Fluoromount aqueous mounting medium (00-4958-02, Thermo Fisher Scientific). Bright-field, phase, and fluorescence images of cells were obtained using an Olympus IX51 inverted light microscope (Olympus, Tokyo, Japan).

### 4.4. Western Blot Analysis

Protein extraction was performed in ice-cold RIPA lysis buffer containing 1 mM phenylmethylsulfonyl fluoride (PMSF; CST-8553S, Cell Signaling Technology) and 1% protease inhibitor cocktail (p2714, Sigma-Aldrich). Cell lysates were incubated for 20 min on ice, centrifuged, and the supernatants were separated on 7.5% SDS-polyacrylamide gel electrophoresis (SDS-PAGE), followed by transfer to nitrocellulose membranes (0.2 μm; PB7320, Thermo Fisher Scientific) using BIO-RAD Mini Trans-Blot Cell. After electrotransfer, the blots were blocked with PBST containing 5% BSA and incubated 1 h at RT with primary antibodies anti-FMRP (BLG-834601, Biolegend) and anti-β-actin (ab8226, Abcam). Blots were then washed with PBST and incubated 1 h at RT with the secondary antibody anti-mouse horseradish peroxidase (CST-7076, Cell Signaling Technology). Blots were detected by enhanced chemiluminescence Western blotting substrate EZ-ECL (RPN2106, Biological Industries) and developed by MYECL Imager (Thermo Fisher Scientific).

### 4.5. RNA Extraction and Quantitative Real-Time PCR

Total mRNA samples were extracted using a Direct-zol RNA miniprep kit (ZR-R2050, Zymo research, Irvine, CA, USA), followed by random hexamer-primed reverse transcription using Superscript IV RT-PCR kit (18091050, Thermo Fisher Scientific). Quantitative real-time PCR (qRT-PCR) was performed using SYBR Green FastMix (95071-012, Quantabio, Beverly, MA, USA). Cycling and analysis were performed using Rotor Gene 6000 Series and its complementary analysis software (v1.7, Corbett, QIAGEN, Düsseldorf, Germany). PCR reactions were performed for three independent experiments with three technical replicates in each experiment. All qRT-PCR assays included a no-template control (NTC) and -RT. GAPDH served as a control to normalize target gene expression.

### 4.6. Magnetic-Activated Cell Sorting (MACS)

At 17 DIV of neuronal differentiation, neuronal cells were dissociated using Accutase to a single cell suspension. Neurons were enriched by the depletion of CD184^+^ cells with the CD184 (CXCR4) MicroBead kit, Human (130-100-070, Miltenyi Biotec Bergisch Gladbach, Germany) following manufacturer’s protocol, and re-plated on PO/L/FN coated plates in NB/B27 supplemented with BDNF, dbcAMP, and ascorbic acid up to 23 DIV.

### 4.7. RNA Sequencing and Bioinformatic Analysis

Total mRNA samples were extracted from hESCs (day 0), at 13 and 23 DIV of their differentiation into cortical neurons, using the mirVana miRNA Isolation Kit (AM9720 + AM1560, Ambion, Austin, TX, USA) according to manufacturer’s protocol. Extraction was performed in three biological experiments. Library preparation and RNA sequencing were performed on Illumina NovaSeq 6000 at a commercial laboratory (Macrogen Inc., Europe, Milan, Italy). Raw sequencing data was trimmed and filtered using fastp 0.19.6 [54], then aligned to the GRCh38 assembly using STAR 2.7.1a [55]. Differential expression analysis was performed using DESeq2 1.24.0 [56] and gene set enrichment was performed using clusterProfiler 3.16.0 [57], both on R version 3.6.3. A heatmap was created using ComplexHeatmap 2.4.232. Taxonomic classification of reads for the contamination was performed using Kraken 2.0.9 [58]. All RNA-seq data from this study can be found in the NCBI Gene Expression Omnibus (GEO) with accession number GSE206088.

### 4.8. Scratch Assay

Neurite outgrowth was measured by the scratch wound assay. Wells were washed with PBS and a cell free area spanning approximately 600 μm in diameter was scratched using a 10 μL pipette tip. The plates were then rinsed with sterile PBS to remove cell debris and replaced with fresh NB/B27 supplemented with 1% Pen/Strep, PD0325901, SU5402, DAPT, BDNF, dibutyryl cAMP, ascorbic acid, and CHIR99021. Treated wells were also supplemented with 10 µM SB431542 and 250 nM LDN-193189, that inhibits the TGFβ- and BMP-mediated activation of SMAD proteins, as well as the phosphorylation of SMADs. The scratch was photographed 0 h and 24 h after its generation: in between, cells were maintained at 37 °C and5% CO2. Images were captured using an Olympus IX71 microscope with a 10X objective using an Olympus IX51 inverted light microscope and CellSence measuring software. Gap width was measured using ImageJ 1.53f51 (NIH, http://rsbweb.nih.gov/ij/ (accessed on 22 September 2020), USA) with the wound healing size tool plugin [59] from three biological experiments, and 30 fields or more were analyzed in each group. Growth was calculated as the difference between the gap width at 0 h and 24 h.

### 4.9. Statistical Analysis and Experimental Design

All statistical analysis was carried out in GraphPad Prism version 8.4.3 (GraphPad Software, Inc., La Jolla, CA, USA). Statistical significance was determined by paired or unpaired two-tailed Student’s t-test or one-way ANOVA. Differences were considered statistically significant when *p* < 0.05.

## Data Availability

The data presented in the study are deposited in the NCBI GEO repository, accession number GSE206088.

## Supplementary Materials

The following supporting information can be downloaded at: www.mdpi.com/article/10.3390/ijms23169278/s1.
