# Supplementary material for: Transcriptomic Analysis of Human Fragile X Syndrome Neurons Reveals Neurite Outgrowth Modulation by the TGFβ/BMP Pathway
Source: Int J Mol Sci. 2022 Aug 17;23(16):9278. doi: 10.3390/ijms23169278 (PMC9409179; doi:10.3390/ijms23169278)
Supplement: Supplementary file 1 [file ijms-23-09278-s001.zip › ijms-1839845-supplementary.pdf]

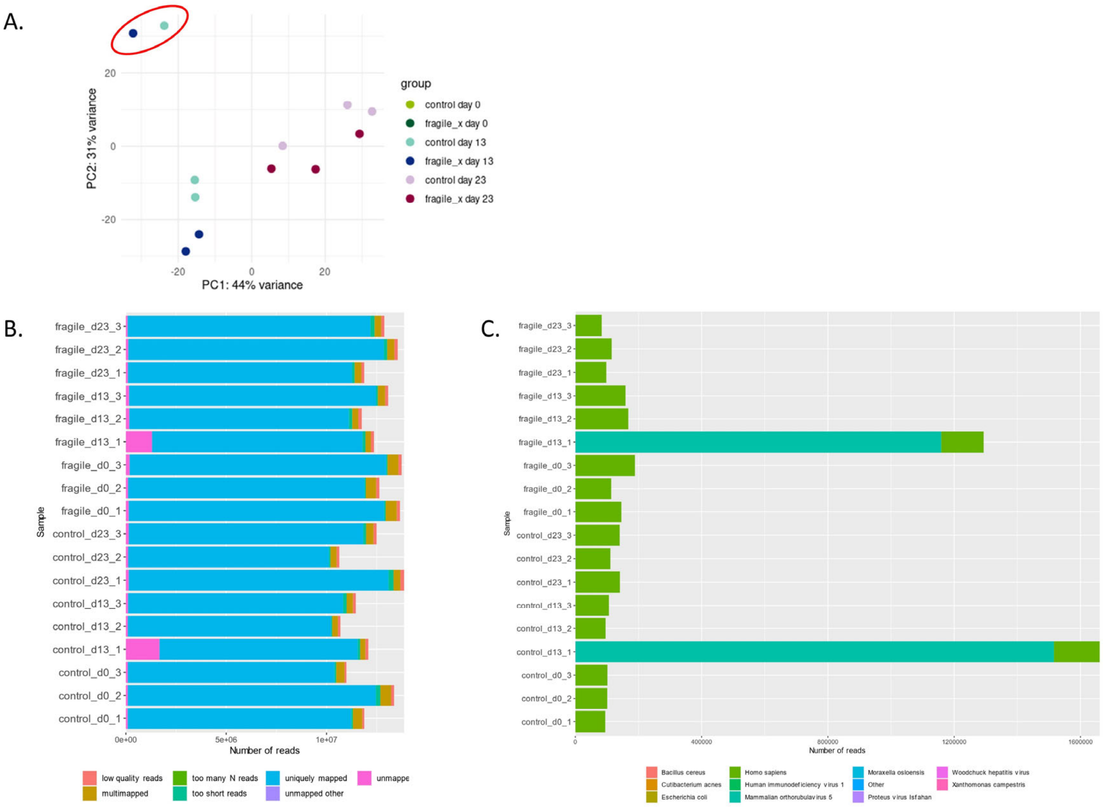

Supplementary Figure S1. Identification of non-human RNA in two of the samples. (**A**) PCA of 13 and 23 DIV samples. Samples suspected of including impure cell population are marked in red. (**B**) Breakdown of sequencing reads by quality and alignment metrics. (**C**) The taxonomic classification of reads that were not mapped to the reference genome. Including reads omitted due to non-unique alignment.

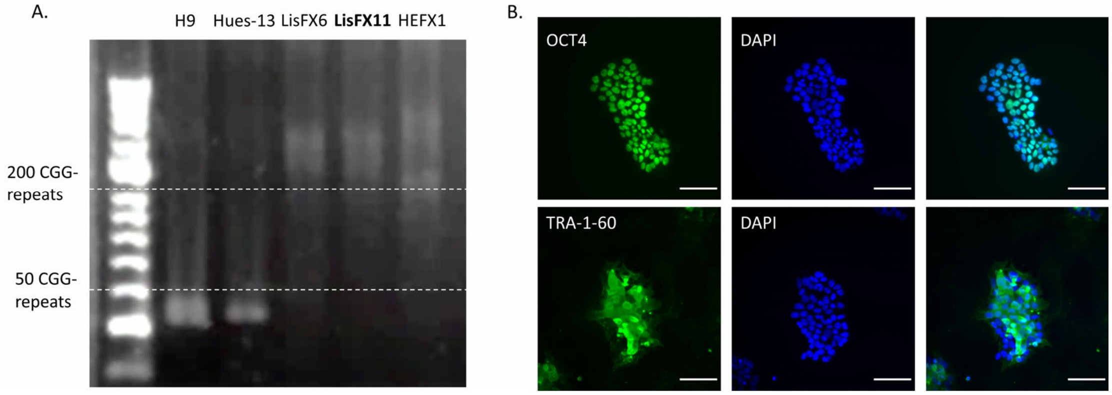

Supplementary Figure S2. Characterization of FX-hESC line (**A**) Analysis of the CGG repeat size by PCR-based repeat-number assay of three different FX-hESC lines used in Fig. 1A. (**B**) Expression of pluripotent genes (OCT4 and TRA-1-60; green) in Lis-FX11. Cell nuclei were stained blue by DAPI. Scale bar: 100 μm.
